# Omics Path to Increasing Productivity in Less-Studied Crops Under Changing Climate—Lentil a Case Study

**DOI:** 10.3389/fpls.2022.813985

**Published:** 2022-05-09

**Authors:** Manish Tiwari, Baljinder Singh, Doohong Min, S. V. Krishna Jagadish

**Affiliations:** ^1^Department of Agronomy, Kansas State University, Manhattan, KS, United States; ^2^National Institute of Plant Genome Research, New Delhi, India

**Keywords:** genomics, lentil, metabolomics, multi-omics integration, proteomics, phenomics, transcriptomics

## Abstract

Conventional breeding techniques for crop improvement have reached their full potential, and hence, alternative routes are required to ensure a sustained genetic gain in lentils. Although high-throughput omics technologies have been effectively employed in major crops, less-studied crops such as lentils have primarily relied on conventional breeding. Application of genomics and transcriptomics in lentils has resulted in linkage maps and identification of QTLs and candidate genes related to agronomically relevant traits and biotic and abiotic stress tolerance. Next-generation sequencing (NGS) complemented with high-throughput phenotyping (HTP) technologies is shown to provide new opportunities to identify genomic regions and marker-trait associations to increase lentil breeding efficiency. Recent introduction of image-based phenotyping has facilitated to discern lentil responses undergoing biotic and abiotic stresses. In lentil, proteomics has been performed using conventional methods such as 2-D gel electrophoresis, leading to the identification of seed-specific proteome. Metabolomic studies have led to identifying key metabolites that help differentiate genotypic responses to drought and salinity stresses. Independent analysis of differentially expressed genes from publicly available transcriptomic studies in lentils identified 329 common transcripts between heat and biotic stresses. Similarly, 19 metabolites were common across legumes, while 31 were common in genotypes exposed to drought and salinity stress. These common but differentially expressed genes/proteins/metabolites provide the starting point for developing high-yielding multi-stress-tolerant lentils. Finally, the review summarizes the current findings from omic studies in lentils and provides directions for integrating these findings into a systems approach to increase lentil productivity and enhance resilience to biotic and abiotic stresses under changing climate.

## Introduction

**Figure 1:**
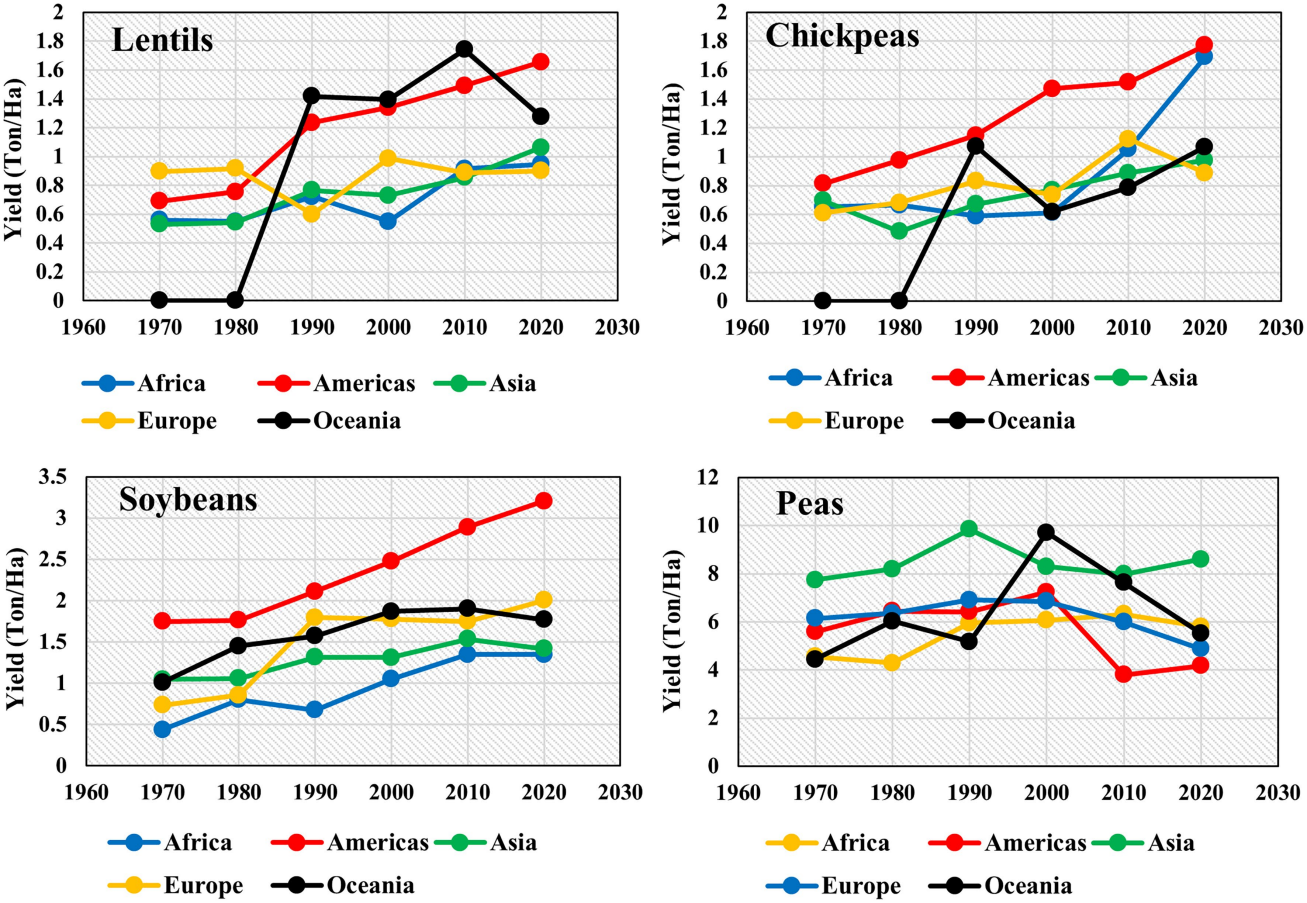
Yield (Ton/Ha) of legume crops lentils, chickpeas, soybeans, and peas at a decadal time scale from 1970 to 2020 in five continents—Africa, Americas, Asia, Europe, and Oceania (FAOSTAT, 2020). FAOSTAT data for lentils and chickpeas were not available for Oceania from 1970 to 1980.

Lentil is a diploid self-pollinated crop having a genome size of ~4 Gbp (Polanco et al., 2018). It serves as a nutritious source of dietary proteins, fiber, minerals, and carbohydrates and reduces malnutrition in developing countries (Erskine et al., 2011). Besides quenching micronutrient deficiency, it is recommended to patients suffering from diabetes, obesity, and cardiovascular diseases because of its low glycemic index (Srivastava and Vasishtha, 2012). Moreover, the cultivation of lentils provides various crop rotational benefits to cereal crops, including symbiotic nitrogen fixation, carbon sequestration, and controlling the proliferation of pests (Kumar et al., 2014, 2020a). Lentils beneficial role in terms of nutrition and ecological sustainability has raised its demand, translating to an increased global lentil production from 0.85 to 6.53 Mt over the past six decades (FAOSTAT, 2020). Globally, lentil productivity is heavily reliant on the amount and distribution of rainfall as lentils are cultivated under rainfed conditions. Early withdrawal of rain results in a low level of residual moisture, and hence residual moisture and change in weather adversely affect germination, growth and determines the final yield (Kumar et al., 2013; Paudel et al., 2020). Excessive rainfall with poor drainage capacity results in waterlogging causing reduced growth and increased risk of fungal infection. Similarly, water-deficit conditions result in poor water availability and limited overall lentil production (Paudel et al., 2020). The crop when faced with drought during the grain-filling stage, and also an abrupt increase in temperature leads to increased senescence. High variability in temperature ranging from low temperature during vegetative and early flowering stages and terminal heat stress are common events in the lentil’s growth cycle in India and Australia (Sehgal et al., 2021). The dependency of lentil on rainfall amount and distribution often leads to low productivity compared to other legumes and crops. The average productivity of legume crops such as lentil, chickpea, soybean, and peas in 2020 across the continents was 1.17, 1.28, 1.95, and 5.8 t/ha, respectively (Figure 1). Other legume crops lag significantly in productivity compared to peas, with lentils being the lowest compared to other legumes.

Although the Asian continent is one of the major lentil consumers, its productivity is the lowest, with yield levels of <1 t/ha (FAOSTAT, 2019). Apart from rainfed cultivation, other reasons for the low yield of lentils are various abiotic stresses such as low soil fertility, heat, cold, terminal drought, and biotic stresses such as collar rot (*Sclerotiun rolfsii*), anthracnose (*Colletotrichum truncatum*), ascochyta blight (*Ascochyta lentis*), stemphylium blight (*Stemphylium botryosum*), fusarium wilt (*Fusarium oxysporum* f.sp. *lentis*), root rot (*Rhizoctonia solani*), rust (*Uromyces viciae-fabae*), and white mold (*Sclerotinia sclerotiorum*; Kumar et al., 2013; Sharpe et al., 2013). During the last four decades, research efforts are focused on assembling and screening diverse germplasm, including promising genotypes in to breeding programs, and improved varieties released with desirable traits (Singh et al., 2018). This has resulted in an increase in lentil yield from 585 to 1,194 kg/ha and production from 1.24 to 5.73 Mt since the 1980s (FAOSTAT, 2019).

Several varieties with improved yield and disease resistance have been released during this duration. In Bangladesh, short-duration varieties such as BARI M4-M8 have increased lentil productivity (Kumar et al., 2021). Shikhar/Shekhar/Shekher (ILL4404), a medium maturing cultivar is currently cultivated in Nepal (Materne and McNeil, 2007). Similarly, in India, Precoz, with large seed size, early seedling vigor, and rust resistance features, has been extensively utilized to develop new, improved cultivars, namely, VL Masoor 507, Angoori, Priya, and Narendra M1 (Kumar et al., 2013). Mainly traditional breeding methods have been used to develop stress-tolerant cultivars with improved yield. However, current annual genetic gains in lentil are <0.7% which is insufficient to meet the growing demand (Kumar et al., 2021). Additionally, traditional breeding strategies have improved lentil varieties for various monogenic traits; however, they are less precise and time-consuming when targeting quantitative traits (Cobb et al., 2019). To enhance and sustain lentil productivity, integration of various omics approaches is considered essential and timely for improving quantitative and environment-controlled traits similar to wheat, rice, and maize (Jiang et al., 2019; Yoshino et al., 2019; Mishra et al., 2021a). Hence, the principal objective of this review is to summarize the current status of lentil breeding programs, research progress achieved using omics technology, and application of these technologies in lentil improvement programs.

To provide a road map for increasing productivity in lentils and to complement ongoing breeding efforts, we have obtained data from various omics studies in lentils and have identified key molecules related to plant development, stress tolerance, and nutritional enhancement. Briefly, we procured the data from the transcriptome, proteome, and metabolome studies and reanalyzed for genes/proteins/metabolites, which are common in two different investigations in a similar type of omics study. Thus, the set of genes/proteins/metabolites which are common between either of the omics technology is marked as putative regulatory molecules of two different biological processes.

## Omics Approaches in Lentil Improvement

“Omics” refers to a package of technologies used to unravel the diversity of molecules in a cell/tissue, such as genes, transcripts, proteins, and metabolites. Highly developed and improved “omics” technologies have exponentially benefited plant systems biology (Bassel et al., 2012). These technological advancements have generated extensive data, and integration of this information will complement breeding strategies. An integrated interactome approach of multi-omics technologies, comprising genomics, phenomics, transcriptomics, metabolomics, and proteomics, can discover the underlying molecular mechanisms controlling plant developmental processes and stress tolerance in lentil. Several investigations in plants report use of multi-omics approaches for better understanding of plant responses at genomic, proteomic, or transcriptomic levels during plant development or different abiotic and biotic stresses (Großkinsky et al., 2018; Crandall et al., 2020; Jiang et al., 2020). New omics-related information is rapidly emerging in lentils, and a similar interdisciplinary approach is required to benefit lentil breeding communities.

## Genomic Innovations Reveal a New Genetic Landscape for Lentil Improvement

So far, classical breeding approaches have contributed to lentil productivity around the world. However, these approaches involve selecting genotypes for desired traits followed by recombination and the final selection of recombinants with improved traits to develop highly productive and resilient cultivars (Britt and Kuppu, 2016). These techniques had limited success for complex quantitative traits (Bernardo, 2020). Many traits of economic importance are quantitative and are often influenced by the immediate microclimate. Thus, genotype–environment interaction poses stiff challenges in the genetic improvement of lentil for quantitative traits by employing traditional methods (Kumar et al., 2015). To overcome these hurdles in crop improvement and development of resilience to adverse environments, a relatively robust and rapid approach of genomics-assisted breeding is widely used (Kole et al., 2015).

Genomics has emerged as a pioneering tool for identifying and selecting high-precision superior alleles and their application in breeding programs through genetic engineering and marker-assisted selection (MAS) to achieve genetic gains in crops (Kole et al., 2015). However, limited efforts have been made toward the implementation of genomics-assisted breeding in lentils. Between 2010 and 2020, advancements in next-generation sequencing (NGS) technologies have led to accelerated development of genomic resources in lentils (Kumar et al., 2015, 2020a). The advent of novel genomic information resulted in the identification of several molecular markers, broadly classified into three classes, i.e., hybridization-based, PCR-based, and NGS-based markers. Initially, hybridization-based first-generation molecular markers such as restriction fragment length polymorphism (RFLP) were used for developing linkage maps in lentils (Kumar et al., 2014). However, the use of these markers was very limited in lentils due to the requirement of highly technical skills for their development.

The emergence of PCR-based markers emanated the second-generation of molecular markers such as random amplified polymorphic DNA (RAPD), sequence characterized amplified regions, amplified fragment length polymorphism (AFLP), inter-simple sequence repeats, and simple sequence repeat (SSR) markers (Grover and Sharma, 2016). These molecular markers are widely distributed across the genome and are used extensively for the construction of linkage maps. The first extensive linkage map in lentils was developed with 177 markers (79 AFLP, 89 RAPD, six RFLP, and three morphological markers) identified from RIL (recombinant inbred lines) populations (derived from a cross between *Lens culinaris* × *Lens orientalis*; Eujayl et al., 1998). Among all the PCR-based markers, SSRs have made a remarkable contribution to the development of lentil linkage maps. The first genomic library generated using genotype ILL5588, led to the identification of 30 polymorphic SSR markers (Hamwieh et al., 2005). Additionally, a set of 122 genomic SSRs were developed for their use in breeding programs (Verma et al., 2014). Development of SSR markers has been augmented in recent years through NGS-based transcriptome analysis (Kaur et al., 2011; Kant et al., 2017). However, breeding programs in lentil rarely use MAS as the genetic maps constructed using RFLP, AFLP, RAPD, and SSR markers lack tight linkage to the genes of interest, and they are limited in their resolution.

To deal with the lack of closely placed, tightly linked markers, and the development of high-resolution linkage maps, NGS-based third-generation molecular markers such as single-nucleotide polymorphisms (SNPs) have been extensively utilized. Various technologies such as kompetitive allele-specific PCR (KASP; Fedoruk et al., 2013; Sharpe et al., 2013), Illumina Genome Analyzer, and Illumina Golden Gate sequencing were performed to identify SNP markers in lentils (Sharpe et al., 2013; Kaur et al., 2014). Apart from these techniques, transcriptome sequencing is also largely explored to identify SNP markers at genic/transcribed regions (Kaur et al., 2011; Verma et al., 2013; Yilmaz Temel et al., 2015; Sudheesh et al., 2016b; Barrios et al., 2017; Singh et al., 2017c, 2019b; Cao et al., 2019; García-García et al., 2019; Morgil et al., 2019; Wang et al., 2020). SNP-based markers have contributed enormously to the construction of linkage maps, analysis of genetic diversity, and trait association (Lombardi et al., 2014; Sudheesh et al., 2016a; Khazaei et al., 2017, 2018; Pavan et al., 2019). Recently, genotyping by sequencing (GBS) method and Diversity arrays technology have been used to identify SNPs and develop high-resolution linkage maps (Ates et al., 2018a; Pavan et al., 2019; Dadu et al., 2021).

Besides generating molecular markers in lentils, significant efforts have been made toward developing RIL mapping populations. The RIL mapping populations play a significant role in identifying genomic regions (QTLs/genes) associated with important agronomic traits such as drought tolerance, winter hardiness, earliness, anthracnose resistance, aphanomyces root rot (ARR) resistance, and nutritionally essential traits such as iron and selenium content (Tullu et al., 2008; Ates et al., 2016; Idrissi et al., 2016; Aldemir et al., 2017; Bhadauria et al., 2017; Ma et al., 2020; Gela et al., 2021b). In addition, QTL mapping has been used to map genomic regions associated with plant development, abiotic stress tolerance, disease resistance, and nutritional parameters. Mane et al. (2020) identified few QTLs from RIL populations of a cross between WA8649090 × Precoz (Table 1). The QTLs were related to plant developmental processes, such as a QTL-hotspot representing six QTLs for shoot length, root length, and seedling length. Moreover, Polanco et al. (2019) identified one QTL each for seed coat spotting, flower color, stem pigmentation, time of flowering in 78 RILs population from a cross between *Lens culinaris* cultivar Alpo, and *Lens odemensis* accession ILWL235. Additionally, three QTLs for seed size were also identified (Polanco et al., 2019). These QTLs associated with plant developmental processes can be used for enhancing plant vigor.

**Table 1:** Summary of QTL studies performed in lentils.

S. No.	Trait	Cross	Population	Markers	Map length	Average marker distance (cM)	QTLs	Phenotypic variation%	References
1	Winter hardiness	WA8649090 × Precoz	106 F6 RILs	175 (RAPDs, ISSRs and AFLPs)	1,192	9.1	Five (winter survival) and four (winter injury)	9.5–28.8	Kahraman et al., 2004
2	Ascochyta blight resistance	ILL5588 × ILL7537 and ILL7537 × ILL6002	F2	72 markers (38 RAPD, 30 AFLP, 3 ISSR and stem pigmentation)	412.5		Five and three	9.3–69.1 and 9.3–33.8	Taylor et al., 2006
3	Plant structure, growth habit and yield	*Lens culinaris* ssp. *culinaris* cv. “Lupa” × ssp. *orientalis* “Boiss”	113 F2	158 (71 RAPDs, 39 ISSRs, 83 AFLPs, and two SSRs)	2,172.4	15.87	23	20–50 and > 80	Fratini et al., 2007
4	Earliness and plant height	Eston × PI320937	108 RILs	207 (144 AFLP, 54 RAPD, and nine SSRs)	1,868	8.9	11 (earliness) and five (height)	5.2–29.2 and 8.9–19.8 respectively	Tullu et al., 2008
5	Leaf area	WA8649090 × Precoz	106 F6 RILs	130 (RAPD, ISSR, and AFLPs)	973		One	20.45	Kahraman et al., 2010
6	Stemphylium blight resistance	ILL-6002 × ILL-5888	206 F7 RILs	139 (21 SSR, 27 RAPD, 89 SRAP, and two morphological markers)	1,565.2	11.6	Three	5.04–45.96	Saha et al., 2010b
7	Ascochyta blight resistance	ILL5588 × ILL5722	94 F5 RILs	196 SSRs/EST-SSRs	1,156.4	7.1	Three (seedling resistance) and three (maturity resistance)	34 and 61	Gupta et al., 2012
8	Seed quality traits (seed shape, color and pattern)	CDC Robin × 946a-46	139 F7 RILs	577 (563 SNPs, 10 SSRs, and four morphological)	697	1.2	13	3.6–39.8	Fedoruk et al., 2013
9	Boron tolerance	Cassab × ILL2024	126 F6 RILs	325 (264 SNPs and 61 SSRs)	1,178	3.7	One (q_boron_IM)	52–71	Kaur et al., 2014
10	Seed weight and seed size	Precoz × L830	126 F8-RILs	216 SSRs	1,183.7	5.48	Two (one qSW and one qSS)	48.4 and 27.5	Verma et al., 2015
11	Flowering time	Precoz × WA8649041	101 F6 RILs	116 RAPD, 23 ISSR, 13 SSR, and 180 AFLP	1,396.3		One	44–60	Kahriman et al., 2015
12	Ascochyta blight resistance	Indianhead × Northfield [IH × NF], Indianhead × Digger [IH × DIG] and Northfield × Digger [NF × DIG]	117, 112 and 114 RILs	406, 329, and 330 (SNPs, SSRs, and EST-SSRs)	1,461.6, 1,302.5 and 1,914.1		Three (IH × DIG) and two (IH × NF)	69 and 52	Sudheesh et al., 2016a
13	Drought tolerance related root and shoot traits	ILL6002 × ILL5888	132 F6-8 RILs	252 (106 SNPs, 13 SSRs and 133 dominant/codominant markers)	2,022.8	8	18	4–28.9	Idrissi et al., 2016
14	Selenium uptake	PI 320937 × Eston	96 RILs	1,784 (four SSRs and 1,780 SNPs)	4,060.6	2.3	Four (SeQTL2.1, 5.1, 5.2, and 5.3)	6.3–16.9	Ates et al., 2016
15	Seed iron concentration	ILL 8006 × CDC Milestone	118 RILs	4,177 SNP	497.1	0.12	21 (FeQTL1.1—FeQTL7.3)	5.9–14.0	Aldemir et al., 2017
16	Seed related traits	WA 8649090 × Precoz	94 RILs	220 (172 RAPD, three ISSR, and 45 SSRs)	604.2	2.74	18 (nine major and nine minor)	>10 and 2.5–9.8	Jha et al., 2017
17	Heat tolerance for seedling survival and pod set	PDL-1 and PDL-2 (tolerant) × JL-3 and E-153 (sensitive)	F2 population	7 SSRs	218.8		Two (qHt_ss and qHt_ps)	12.1 and 9.23	Singh et al., 2017b
18	Anthracnose and stemphylium blight resistance	L01-827A × IG 72815	94 F9 RILs	2,180 SNPs	740.94	1.36	11 QTLs for anthracnose and three QTLs for stemphylium blight	8.89–24.75	Bhadauria et al., 2017
19	Manganese uptake	CDC Redberry × ILL7502	120 RILs	5,385 SNPs	973.1	0.18	Six QTLs (MnQTL1.1–7.1)	15.3–24.1	Ates et al., 2018b
20	Milling quality (dehulling efficiency, milling recovery, and football recovery)	CDC Robin × 946a-46	127 F7 RILs	545 (534 SNPs, seven SSRs, and four morphological)	697	1.2	Multiple qtls	6.3–34.74	Subedi et al., 2018
21	Boron tolerance	ILL2024 × ILL6788	178 F6 RILs	758 (731 SNPs and 27 SSRs)	1,057	2	One	76	Rodda et al., 2018
22	Multiple agronomical traits	*L. culinaris* cv. Alpo × *L. odemensis* accession ILWL235	78 F7 RILs	6,306 SNPs and short indels	5,782.19		10 QTLs (1—seed coat spotting, 1—Flower color, 1—stem pigmentation, 1—Time of flowering, 3—Seed size and 3—ascochyta resistance)	18.63–85.07	Polanco et al., 2019
23	Salinity stress tolerance at seedling stage	L-4147 × PDL-1	F2 population	Seven SSRs	133.02		One (qS_ss)	65.6	Singh et al., 2020
24	Aphanomyces root rot	K192-1 × K192-2	189 F6 RILs	2,865 SNPs	978.1		19	5.2–12.1	Ma et al., 2020
25	Early plant vigour	WA8649090 × Precoz	94 F10 RILs	265 (90 SSRs, three ISSRs, and 172 RAPDs)	809.4	3.05	14	9.2–21.4	Mane et al., 2020
26	Malate secretion	L-7903 × BM-4	146 F6 RILs	10 AI resistance linked SSRs	138.3		One (qAlt_ma)	60.2	Singh et al., 2021a
27	Flowering time	*Lens culinaris* cv. Lupa and *L. orientalis* accession BGE 016880	93 RILs	4,073 SNPs	5,923.3	1.5	13	17–62.9	Yuan et al., 2021
28	Anthracnose resistance	*L. culinaris* Eston × *L. ervoides* IG 72815	168 F7 RILs	5,455 SNPs	3,252.8	0.6	Two (qANTH-3 and qANTH-7)	8.3–31.2	Gela et al., 2021b
29	Ascochyta blight (AB) resistance	ILWL 180 × ILL 6002	140 RILs	2,514 SNPs	545.4	0.27	Seven (four associated with leaf lesion score, two linked to stem lesion score, and one linked to AUDPC)	9.5–11.5	Dadu et al., 2021

Lentil, due to its dryland cultivation, is exposed to a wide range of abiotic stresses, and hence, its inherent yield potential is not entirely realized, leading to low productivity. In addition to low productivity, the availability of genomic information is equally limited, emphasizing the timeliness of increasing investigations on abiotic stress tolerance to develop tolerant cultivars. In this direction, drought stress-associated QTL analysis was conducted in a RIL population (ILL6002 × ILL5888) which revealed 18 QTLs associated with 14 root and shoot traits such as dry root biomass, lateral root number, specific root length, root-shoot ratio, and chlorophyll content (Idrissi et al., 2016). Similarly, a QTL associated with seedling survival under salinity stress was identified in a population developed by crossing salt-tolerant (PDL-1 and PSL-9) and salt-sensitive (L-4147 and L-4076) genotypes (Singh et al., 2020). Lentils, based on their geographic location, and being a temperate legume are often exposed to very low-temperature conditions, and hence, it is imperative to look for QTL associated with winter hardiness. Five independent QTLs associated with winter survival were identified in 106 RILs population derived from a cross between WA8649090 (winter hardy) and Precoz (non-hardy; Kahraman et al., 2004). One of the QTL showed consistent expression throughout different winter environments and hence was proposed as a desirable QTL for MAS.

Over the past two decades, the exponential leap in identification of molecular markers has resulted in an accurate detection of QTLs inducing stress resilience. Further, the markers are fine-tuned with appropriate breeding strategies for developing and releasing tolerant/resistant varieties with enhanced durability (Pilet-Nayel et al., 2017). Lentil has also been explored for biotic stress-associated QTLs but to a minimal extent, including 14 QTLs reported in *Lens ervoides* for resistance against *Colletotrichum lentis* and *S. botryosum* (Bhadauria et al., 2017). In addition, Sudheesh et al. (2016a) identified five QTLs linked to resistance against *A. lentis*, from RIL populations between IH (Indianhead) × DIG (Digger) (112 RILs and two QTLs) and IH × NF (Northfield) (117 RILs and three QTLs). Similarly, Dadu et al. (2021) identified seven QTLs from a biparental population obtained from a cross between *A. lentis*-resistant accession ILWL 180 (*Lens orientalis*) and a susceptible cultivar ILL 6002. Of these seven QTLs, four QTLs were associated with leaf lesion score, two to stem lesion score, and one to area under disease progress curve (measure of disease progression over time). Moreover, Polanco et al. (2019) mapped three QTLs associated with *A. lentis* resistance in a biparental RIL population obtained from a cross between *L. culinaris* cv. Alpo and *L. odemensis* accession ILWL235.

A dynamic equilibrium exists between mineral uptake from soils, their distribution, and accumulation in different plant parts including seeds. Besides mapping QTLs associated with plant developmental processes, investigations in lentil’s mineral ion uptake resulted in identifying four QTL regions and 36 putative QTL markers related to selenium uptake (Ates et al., 2016), and six QTLs associated with manganese uptake (Ates et al., 2018b). These QTL studies lay the foundation for the future development of micronutrient-enriched lentils with enhanced biofortification. Genome-wide association studies (GWAS) have been extensively conducted to explore genomic regions associated with agriculturally important traits as well as stresses in several crop species, such as wheat (*Triticum aestivum* L.), maize (*Zea mays* L.), rice (*Oryza sativa* L.), sorghum [*Sorghum bicolor* (L.) Moench], soybean [*Glycine max* (L.) Merr.], barley (*Hordeum vulgare* L.), cotton (*Gossypium hirsutum* L.), and the model plant species *Arabidopsis* (Tibbs Cortes et al., 2021). However, GWAS application in lentil is very limited with studies mainly restricted to traits such as carbohydrate, iron, zinc content, anthracnose, ascochyta, *aphanomyces* resistance, and agronomic traits (Table 2; Singh et al., 2017a; Khazaei et al., 2018; Kumar et al., 2019; Ma et al., 2020; Dadu et al., 2021; Gela et al., 2021a; Johnson et al., 2021). GWAS studies are urgently needed in lentils to understand complex traits related to abiotic and biotic stress, plant development, and nutritional aspects. A prerequisite for modern breeding is widely available reference genome assembly to compare between different populations for allelic variants identification, mapping, and correlation with phenotypic variation. Besides other genomic resources, a draft genome assembly of lentil cultivar CDC Redberry is available.^1^ The draft assembly consists of seven pseudomolecules having a size of 4.3 Gb (Bett et al., 2016). This genomic resource can be utilized in lentil breeding programs to identify markers linked to agronomic traits and several important gene families that are widely explored in other plant species (Mishra et al., 2021b; Tiwari et al., 2021b,d). Overall, genomic resources have helped in lentil improvement through genetic finger printing and diversity analysis (Hamwieh et al., 2009; Kumar et al., 2014) and the determination of hybrid status of the crosses between diverse parents (Solanki et al., 2010). Additionally, genomics assisted in the identification of molecular markers associated with desirable gene(s)/QTL for MAS (Kumar et al., 2015), GWAS mediated marker-trait association analysis in the diverse natural populations (Fedoruk et al., 2013), and genetic transformations with the gene of interest (Khatib et al., 2007, 2011). For increased precision and efficient identification and selection of superior recombinants, functional genomics, i.e., integration of MAS accompanied with genetic engineering can be employed in lentil breeding programs. This will lead to the introgression of favorable genetic variability in the cultivated gene pool.

**Table 2:** Summary of genome-wide association studies (GWAS) studies performed in lentils.

S. No.	Trait	Accessions	Total markers used in study	Associated markers	Phenotypic variation	Reference
1	Iron and zinc concentration	138	1,150 SNPs	Two SNPs for iron and one SNP for zinc	9–21%	Khazaei et al., 2017
2	Iron and zinc concentration	96	73 SSRs	Three SSRs (PBALC 13, PBALC 206, and GLLC 563) for iron and four SSRs (PBALC 353, SSR 317–1, PLC 62, and PBALC 217) for zinc	9–11% and 14–21% respectively	Singh et al., 2017a
3	Seed quality traits (seed diameter, thickness, and plumpness)	138	1,150 SNPs	Six Marker-trait associations		Khazaei et al., 2018
4	Grain diameter and weight	96	425 (365 EST-SSRs and 60 genomic markers)	Three (PBALC 224—grain diameter and GLLC-614 and PBALC 29—grain weight)	7–15%	Singh et al., 2018
5	Agronomic traits (plant height, days to flower, days to maturity, seeds per pod, 100 seed weight, biomass yield, seed yield, and harvest index)	96	534 SSRs	28 Marker-trait associations for nine traits	7.3–25.8%	Kumar et al., 2018b
6	Flowering time	96	75 SSRs	26 SSRs	2.1–21.8%	Kumar et al., 2018a
7	Iron and zinc concentration	96	80 SSRs	Two SSRs (GLLC 106 and GLLC 108) for iron and three SSRs (PBALC 364, PBALC 92, and GLLC592) for zinc	17 and 6% (iron) and 6, 8 and 13% (zinc)	Kumar et al., 2019
8	Aphanomyces root rot	326	4,558 SNPs	38 QTLs	1.4–21.4%	Ma et al., 2020
9	Carbohydrate content	143	22,222	Multiple SNPs	-	Johnson et al., 2021
10	Anthracnose resistance	200	1,52,011 SNPs	14 SNPs	58–69%	Gela et al., 2021a

Replacement of traditional lentil landraces worldwide with systematic bred genetically uniform cultivars may result in loss of genetic diversity (Khazaei et al., 2016). Therefore, lentil breeding can also benefit from preserving lentil genetic resources as an agricultural legacy in form of geneBanks. Not only seed storage and plant propagation is required but also a strategy to identify and transfer desired alleles from wild relatives to modern varieties is also needed. Genomic sequence information can be used as molecular passport at single plant level and this may serve as a bio-digital resource for selection of genetic variants and their use in breeding programs (Mascher et al., 2019). Major challenges that lentil geneBank can face are (i) tracking accessions identity, (ii) maintenance of accessions genetic integrity, and (iii) circumventing redundancy within and between geneBanks (Mascher et al., 2019). A recent surge in availability of high-throughput genotyping data can yield ample number of SNPs to demarcate each accession. Hence, dense genotyping information can serve as molecular passport to demarcate accessions. An innovative and modern way for tracking accession identity by irreversibly and immutably storing data across agricultural system can involve use of blockchain technologies (Demestichas et al., 2020). Blockchain is a digital distributed ledger used to incorporate a constant growing list of data records. So blockchain augmented with artificial intelligence and machine learning can be seen as a future to support sustainable and equitable agriculture.

Although, the application of genomics in lentil has fast paced recently, breeding approach still requires (i) application of available genomic resources such as QTLs, linkage maps, and association studies in lentil breeding programs as performed in other legume crops such pigeonpea and chickpea (Roorkiwal et al., 2020), (ii) several QTLs have been reported in lentil related to different agronomically important traits; however, efforts toward fine mapping of those QTLs are limited, and (iii) markers related to several traits such as rust, stemphylium blight, and selenium uptake are available that could be utilized in marker-assisted breeding programs. However, the pace of development of such markers with tight linkage in very slow as compared to other legumes (Saha et al., 2010a,b; Ates et al., 2016; Dikshit et al., 2016; Singh et al., 2021b). There is a need to identify tightly associated markers for other important traits that could be utilized in MAS for lentil yield improvement.

## High-Throughput Phenomics to Capitalize on Advances in Genomics

Although significant progress has been achieved with advances in genomics leading to an improved understanding of mechanisms, translating this progress to crop improvement has not been achieved. The reason partly lies in obtaining large-scale spatial and temporal phenotypic datasets and correlating the genome to the phenome (Mir et al., 2019). Therefore, the real bottleneck has shifted from the knowledge in genomics to a gap in generating high-quality phenomics information on large populations. Phenomics is a systematic, high-throughput refinement, and rapid characterization of a phenotype involving quantitative and qualitative traits such as morphological, biochemical, physiological, and imaging techniques. Plant phenomics mainly includes examining the structure and function of plants (Tardieu et al., 2017). Thus, phenomics not only deals with the association of a genotype and corresponding phenotype but also the characterization of plasticity of phenomes upon exposure to varying environmental conditions and their interactions (Pieruschka and Schurr, 2019). Although phenomics provides an exciting route to understand the behavior of plants to environmental interactions based on genetic backgrounds, applying phenomics technologies to lentils has been challenging. The reason is largely because lentils treated with stress under controlled conditions show a completely different phenomenon than plants under stress in field conditions (Kumar et al., 2020b). Therefore, it is recommended to apply phenomics to lentil studies under field conditions.

Several modifications have been made in conventional manual phenotyping approaches to capture the physiological responses in lentils. A conventional physio-molecular approach includes applying high-precision laboratory techniques to measure different physiological traits such as membrane stability, photosynthetic rate, pollen/ovule germination and viability, and pod set in field grown lentil to differentiate heat tolerant from susceptible genotypes (Kumar et al., 2016; Sita et al., 2017; Chaudhary et al., 2020). Additionally, a different technique, i.e., the focused identification of germplasm strategy (FIGS), employs the concept that the interaction with harsh environments helps identify heat-tolerant genotypes (Delahunty et al., 2015; Gaur et al., 2015). This technique relies on the rationale that lentil accessions grown under heat-stress prone locations would have a greater probability of harboring traits and genes responsible for heat tolerance.

Sensor-based phenotyping methods are currently replacing these conventional screening methods. Recent development in phenotyping technologies includes thermal imaging, multi/hyperspectral sensors, digital Red-Green-Blue (RGB) imaging, and fluorescence scanning (Mahlein, 2016). These are further augmented with improvements in pattern recognition and machine-learning approaches, thus leading to the phenotyping of thousands of plants at a rapid pace with increased precision (Singh et al., 2016). These approaches primarily use imaging techniques to discern minute differences in plant responses which are almost impossible to capture by visual assessment (Marzougui et al., 2019). Dissanayake et al. (2020) developed high-throughput phenotyping (HTP) method for screening lentils under different stresses including salinity. For standardization, six lentil genotypes were exposed to different salt concentrations (0.0, 42.5, 85.0, or 127.5 mmol, NaCl) and subsequently digitally RGB imaged at 10 days after germination and every alternate day for 2 months following salt treatment. After standardizing the method under glass house conditions, they applied HTP protocol to screen 276 accessions, with the same set previously assessed using the conventional phenotyping method (Dissanayake et al., 2020). Comparison of salt tolerance scores obtained after conventional screening and image-based assay revealed a moderate correlation (*r* = 0.55; *p* < 0.0001) and significant correlation upon validation by Spearman rank correlation analysis (*r* = 0.68; *p* < 0.0001). Interestingly, CIPAL1522, lentil line was scored as tolerant under traditional phenotyping, whereas moderately tolerant in the HTP method (Dissanayake et al., 2020). The comparative analysis between the HTP and conventional screen through detailed phenotypic trait assessments revealed improved precision and superior consistency using HTP compared to conventional phenotyping.

Currently, assessments are primarily limited to counts or rating estimates of disease severity and concomitant consequences on plant productivity (Pilet-Nayel et al., 2002). The routine screening process for disease severity usually involves large populations. Therefore, the conventional phenotyping methods using counts or scoring are time-consuming, laborious, and rely heavily on the researcher’s expertise (Adu et al., 2014). To overcome these limitations, HTPs were used to evaluate resistance to ARR caused by *Aphanomyces euteiches* in lentil genotypes in a greenhouse (using hyperspectral and digital RGB imaging) and field condition (using unmanned aerial system-based multispectral imaging; Marzougui et al., 2019). Early infection of root rot occurs in the root, and the aboveground shoot remains asymptomatic; hence, it is challenging to score disease severity during the seedling stage (Ma et al., 2020). However, the root features such as projected area, convex hull area, perimeter, compactness, solidity, major and minor axis lengths analyzed using RGB images showed a strong association with disease severity. The results indicated that lentil breeders can successfully adopt image-based phenotyping approaches to objectively quantify resistance to select potential tolerant donors with increased precision.

Although, the phenotyping techniques have diversified a lot from visual screening to HTPs, still application of modern phenotyping approaches is lacking in lentil. Phenotyping lentils face stiff challenges similar to other crops, such as intertwining of neighboring plants and effect of environment on sensor accuracy, resolution, and data collection (Fernandez et al., 2017). Therefore, to cope with these limitations, lentil phenotyping needs highly flexible non-destructive robotic measurement platforms, with precise navigation systems, diverse sensor modules compatibility, and potential to simultaneously evaluate multiple plots with different data formats (Fernandez et al., 2017). In soybean and wheat, a multisensor phenotyping system comprising of integrated infrared radiometers, ultrasonic distance sensors, NDVI (normalized difference vegetation index) sensors, RGB camera, and portable spectrometers was employed to quantify canopy traits (Bai et al., 2016). A high correlation between the sensor-, final grain yield, and canopy-based traits was observed. Thus, a similar system can provide excellent results for lentil crop breeding and phenotyping. Although, the multisensor system worked well for phenotyping, still the results suffered discrepancies upon facing environmental variations, such as light intensity, humidity, wind, and mechanical vibration (Bai et al., 2016). In summary, a comparative and integrated examination of genetic and phenotypic variability related to traits of interest, their conservation, and influence on the fitness of crop in response to different stresses are fundamental to define the potential vulnerability of lentils and opportunities to select resilient genotypes.

## Transcriptome Profiling Reveals Hidden Gene Regulatory Networks

Genomics and phenomics approaches reveal significant information on genotype and corresponding phenotype. However, genes engaged in regulatory networks and their mechanism of action remains elusive (Martin and Wang, 2011). Plants employ transcriptional reprograming to synchronize growth and development and for providing stress endurance (Martin and Wang, 2011). Investigations revealing the complete set of transcripts ranging from large coding RNAs, small RNAs, long non-coding RNAs, circular RNAs, novel transcripts, and alternatively spliced forms to gene-fusion transcripts constitute the broad area of transcriptomics. Transcriptomics is fundamental and comparatively economical among all omics strategies and, hence, most widely used till date (Dumschott et al., 2020; Jung et al., 2020). The advent of NGS has further reduced the cost, accompanied with increased accuracy (Kant et al., 2017; Tripathi et al., 2018; Tiwari and Bhatia, 2020; Tiwari et al., 2021a,c). Various transcriptomic studies have been performed to get an insight into diverse functional aspects of lentil plants such as growth, development, response to stress, and marker identification, development, and application (Kaur et al., 2011; Verma et al., 2013; Yilmaz Temel et al., 2015; Sudheesh et al., 2016b; Barrios et al., 2017; Singh et al., 2017c, 2019b; Khorramdelazad et al., 2018; Cao et al., 2019; García-García et al., 2019; Morgil et al., 2019; Wang et al., 2020).

Considering the challenges from abiotic stresses, transcriptomic studies were performed in lentils for understanding intricate nexus among genes to provide tolerance against abiotic stresses such as heat, drought, and cold. Singh et al. (2019b) conducted a transcriptome analysis for heat-stress treatment using tolerant PDL-2 and sensitive JL-3 lentil cultivars grown at 27/16°C (day-night) for optimal conditions and 35/33°C continuously for 3 days as heat stress. Their comparative analysis led to the identification of 13,510–16,817 heat-responsive DEGs. Additionally, 141,050 microsatellites, 194,178 high-quality SNPs, and 7,388 insertions-deletions (Indels) were also established. The number of DEGs in tolerant PDL-2 was higher in contrast to sensitive genotype, and most of the DEGs belonged to cell wall/callose deposition enzymes (Plasmodesmata Callose-Binding Protein 3), wax formation (Diacylglycerol acetyltransferase WSD1), secondary metabolic processes (Pyruvate phosphate dikinase chloroplastic), and several transcription factors (WRKY and NAC; Singh et al., 2019b). The cell wall modifying enzymes such as Pectin Methylesterase (PME) help plants cope with abiotic stress and callose accumulation and have been established as a protective mechanism against biotic and abiotic stress by plasmodesmata regulation and signaling events (Chen and Kim, 2009). Heat stress activates transcription of PME, and apoplastic Ca^2+^ mobilization resulting in cell wall remodeling ultimately leading to thermotolerance (Tiwari et al., 2022). After heat stress exposure, cell wall stiffening occurs during the recovery period, where PME mediated demethylesterification and interaction with Ca^2+^ leads to an aggregated pectate gel lawn (Wu et al., 2018).

Heat stress is often accompanied with water-deficit conditions, and these two factors, independently or in combination, frequently limit the growth and productivity of lentils (Sehgal et al., 2017; Rane et al., 2022). Although lentil is moderately tolerant to drought, intermittent and terminal drought stress negatively impacts productivity during the grain filling (Sánchez-Gómez et al., 2019). To get an insight into the drought-responsive transcriptional circuitry-, short-, and long-term drought stress (1 and 4 days, respectively) was imposed on a drought-sensitive cultivar “Sultan.” Short-term drought stress led to the identification of 2,915 DEGs in the root tissue, whereas the number increased exponentially to 18,237 under long-term drought stress (Morgil et al., 2019). Another drought stress study conducted on the resistant PDL-2 and sensitive cultivar JL-3 compared to control, revealed 11,435 upregulated and 6,934 downregulated transcripts (Singh et al., 2017c). Interestingly, analysis of DEGs revealed that genes involved in the TCA cycle, transporters category, respiratory electron transport chain, ABC family, and glucose metabolism were upregulated in sensitive and tolerant genotypes compared to respective controls. However, comprehensive downregulation in the expression of genes involved in photorespiration, photosynthesis, and carbohydrate metabolism was observed in the tolerant than the sensitive genotype (Singh et al., 2017c; Morgil et al., 2019). Since drought tolerance is an outcome of intricate molecular mechanisms associated with several genes, detailed investigations focusing on the most promising target genes are needed (Rane et al., 2022).

Lentil is a temperate legume with frost endurance capabilities of low temperature up to −6°C (Cash et al., 1996). Early or winter sowing increases crop yield by 20–100%; hence, cold tolerant cultivars are desired (Mikić et al., 2011). Thus, breeding programs have majorly focused on developing cultivars with increased cold tolerance to improve lentil productivity. Capturing gene expression profiles during cold stress will provide an insight into the underlying gene regulatory network during the stress period. Barrios et al. (2017) annotated RIL populations from a cross between frost susceptible, Precoz, and tolerant WA8649041 genotypes as cold tolerant or susceptible based on their response to freezing temperature ranging between −3 and −15°C. Simultaneously, a Deep Super-SAGE transcriptomic analysis was conducted on eight tolerant and seven sensitive RILs along with respective controls. SAGE analysis identified ~300 differentially expressed tags which mainly encode dormancy-related proteins, glycine-rich, cold and drought-regulated proteins, membrane proteins, and proline-rich proteins (PRPs). The molecular and biochemical mechanisms underlying cold acclimation majorly involved differential expression of cold-responsive genes, altered photosynthesis, remodeling of cell membranes, activation of reactive oxygen species, and antioxidants. These results point toward the differential expression of genes related to transmembrane proteins and photosynthetic functioning in lentils to achieve cold acclimation.

Rainfed winter-grown food legumes experience high humidity and face significant challenges to sustain yield potential due to increased foliar and root rot diseases (Solh et al., 1994). Among biotic stresses, foliar diseases cause significant loss to lentil yield. Ascochyta blight is one of the major devastating seed or stubble-borne foliar diseases and leads to yield reduction ranging between 30 and 70% in Canada, Australia, United States, and India (Singh et al., 2017d). Lentil is also prone to several other pathogens, resulting in diseases such as stemphylium blight, anthracnose, lentil rust, sclerotinia white mold, and botrytis grey mold (*Botrytis fabae* and *B. cinerea*; Garkoti et al., 2013). To identify responses of lentil cultivars against biotic stresses, transcriptomic studies focusing on major foliar diseases caused by *A. lentis* and *S. botryosum* have been performed. Transcriptional landscape of resistant (ILL7537) and susceptible (ILL6002) lentil genotypes against *A. lentis* infection at 2, 6, and 24 hours post-infection (hpi) was recorded (Khorramdelazad et al., 2018). The study revealed a primary response at 2 hpi with significantly higher expression of most of the pathogen recognition and defense-related genes such as LRR receptor-like kinases (LRR-RLKs) and Calmodulin domain protein kinase-like (*CDPK*). Gene expression pattern indicated a very early detection of pathogenic microbial invasion and activation of the pathogen-associated molecular pattern (PAMP)-triggered immunity (PTI). Downstream of kinases, calcium spiking signaling cascade is initiated to defend against the infection (Khorramdelazad et al., 2018; Thapa et al., 2018; Mishra et al., 2021b; Tiwari et al., 2021b). Further, gene enrichment of DEGs revealed secondary defense response at 6 hpi in the form of hyperactivity of transcriptional regulators, production of antifungal compounds, and construction and reorganization of cell walls. A tertiary defense response was employed at 24 hpi, where the functional attributes shifted to activation of mechanisms associated with stress tolerance, photosynthetic pathways, and antimicrobial compounds.

Stemphylium blight is another foliar disease caused by fungi and characterized by initial tan to light brown spots on leaves, which later spread to all parts including, leaves, stem, flowers, and seeds (Mwakutuya and Banniza, 2010), resulting in up to 80% economic loss (Cao et al., 2019). *Lens ervoides* RILs, LR-66-577, and LR-66-637 were annotated as susceptible and resistant, respectively, based on a high percentage of conidial germination, penetration, and progressive necrosis in LR-66-577 compared to LR-66-637 upon *S. botryosum*, SB19 infection (Cao et al., 2019). The response of *Lens ervoides* cultivar to stemphylum blight disease was examined using transcriptome of LR-66-637 and LR-66-577 at 0, 48, 96, and 144 hpi (Cao et al., 2019). The investigation led to the identification of 8,810 disease-responsive genes and 1,284 DEGs comprising 712 upregulated genes in the resistant and 572 in susceptible RILs (Cao et al., 2019). Interestingly, the comparison of DEGs present during *A. lentis* and *S. botryosum* infection showed several common genes related to defense signaling and phytohormones such as E3 Ubiquitin protein, LRR-RLKs, CDPK, and auxin repressed protein. The presence of recurrent DEGs is indicative of a common mechanism employed by distinct lentil cultivars to provide resistance to these two different pathogens.

**Figure 2:**
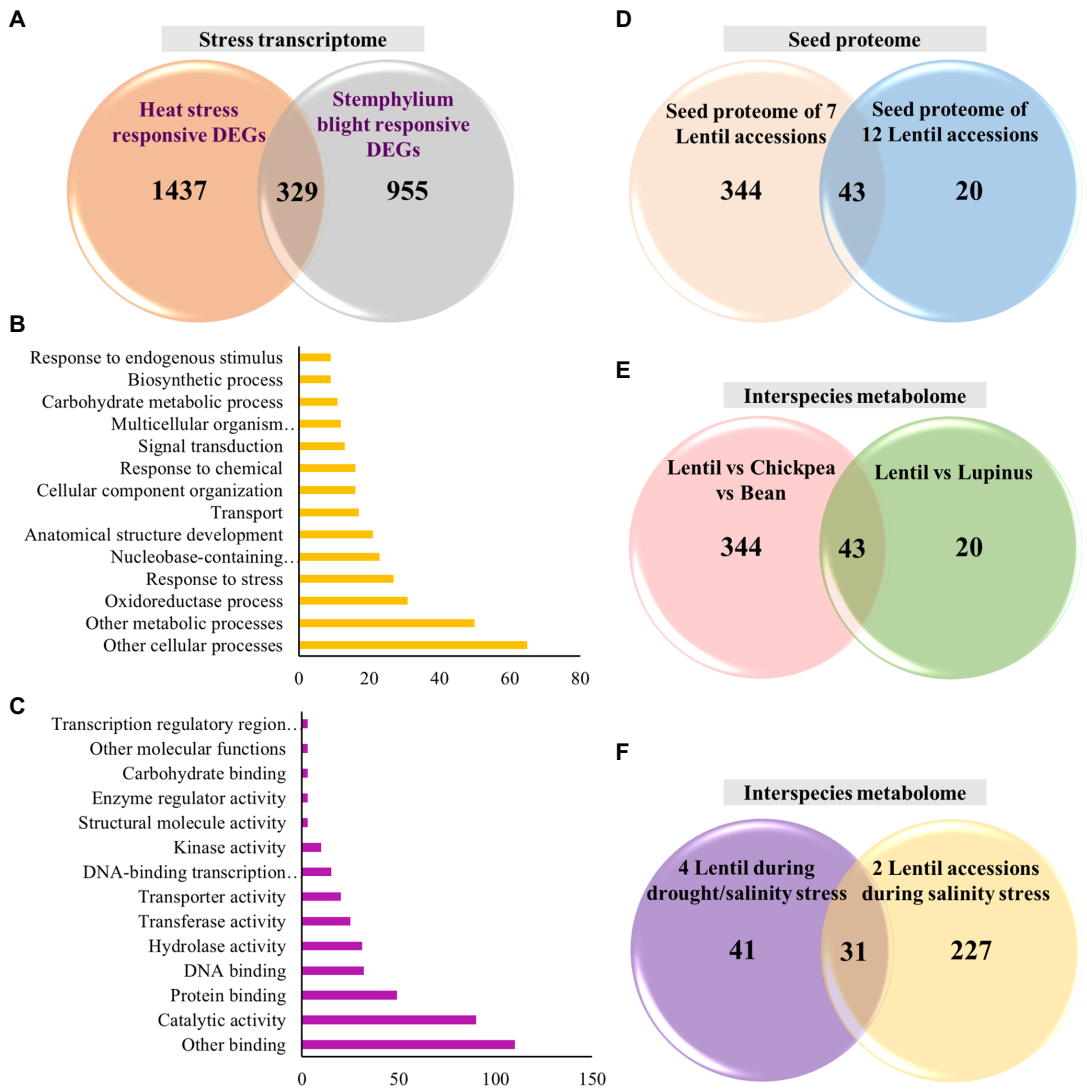
Venn diagrams representing distribution of genes/proteins/metabolites in different lentil studies and gene ontology (GO) functional analysis of DEGs. **(A)** Distribution of DEGs during heat stress and stemphylium blight infection and intersection shows common set of DEGs; **(B)** the most significantly enriched GO terms in biological process; **(C)** the most significantly enriched GO terms in molecular function; **(D)** distribution of identified proteins obtained from lentil seed proteome and intersection shows common set of proteins; **(E)** distribution of metabolites in interspecies metabolite studies and intersection shows common set of metabolites; and **(F)** distribution of metabolites in intraspecies lentils metabolite studies during salinity/drought and drought stress and intersection shows common set of metabolites.

Further, in this review, we performed an analysis for identifying the common set of DEGs involved in biotic and abiotic stresses. For this analysis, we obtained the heat (tolerant vs. sensitive, 6,284 transcripts) and stemphylium blight (resistance vs. susceptible, 8,810) responsive transcripts from respective transcriptome studies (Cao et al., 2019; Singh et al., 2019b). To find the DEGs, from these studies, we narrowed down to transcripts with fold change > 2 or FDR < 0.05 resulting in 1,766 DEGs during heat stress and 1,284 during stemphylium blight infection (Figure 2A). We identified 329 common transcripts across these studies. The common set of DEGs included kinases such as LRR-RLKs and serine/threonine kinases, phytohormone auxin and ABA related genes, cell division and cell cycle control genes, secondary metabolites, cell wall forming and maintenance genes, defense-related, photosynthetic pathway-related, heat shock proteins and transcription factors, and others (Supplementary Table 1). Additionally, gene ontology (GO) analysis of the DEGs revealed that among biological processes the genes were involved in cellular and metabolic processes as well as oxidation–reduction process (Figure 2B). Among the molecular function, the genes were enriched in several binding activities including DNA, RNA, and metal ion binding as well as catalytic activities (Figure 2C). These studies provide insights on genes involved in crosstalk between heat and biotic stress.

Besides apprehending the transcriptomic landscape involved in biotic and abiotic stresses, transcriptomic studies also identified information on markers such as SNPs, SSRs, and KASP. Transcriptome analysis in six lentil accessions annotated 26,449 EST-SSR markers, of which 276 were screened and were utilized to check polymorphism in 94 accessions (Wang et al., 2020). Of the 276 markers, 125 were found to be polymorphic. Additionally, 130,073 SNP loci were detected, and of these, 127 were picked for KASP marker development. Out of 127 SNPs, 78 were successfully converted to KASP markers (Wang et al., 2020). Similarly, Kaur et al. (2011) also performed cDNA sequencing analysis on six lentil genotypes and reported 2,393 distinct loci of EST-SSR markers. Among these, 192 were screened across 13 lentil genotypes, which showed 47.5% genetic polymorphism. Another study reported 8,722 SSRs, of which 96 primer pairs were validated by PCR. Fifty-four of the amplified primers were selected for diversity analysis, resulting in a total of 23 (42.6%) polymorphic SSRs (Verma et al., 2013). The EST-SSR, SNP, and KASP markers obtained in these studies are essential tools for linkage mapping and comparative genomics, playing a crucial role in molecular marker-assisted breeding of lentils. Besides examining genetic diversity, these markers can also be used for accurate and rapid detection of seed purity and the authenticity of seeds. Traditional distinctness, uniformity, and stability (DUS) evaluation are conducted with morphology descriptors, and the results could be confounded because of the duration of the test period and the environment (Idrissi et al., 2019). Molecular markers have emerged as the frontrunners for rapid, cost-effective, accurate testing, and characterization of genotypes/cultivars through DNA fingerprinting and genetic diversity analysis (Li et al., 2019). Thus, DNA marker technology has empowered lentil geneticists/breeders in understanding genomic architecture and molecular detection of distinct varieties. A complete information of the transcriptome studies performed in lentils is provided in Table 3.

**Table 3:** Summary of transcriptomic studies performed in lentils.

S. No.	Cultivar/Genotype	Tissue/Stress/Time points	Number of libraries	Sequencing platform	Contigs assembled	DEGs	SSR/SNP markers	Findings	Reference
1	RILs (Precoz x WA8649041)	Two-leaf stage seedling leaves/cold/6°C-12 h light 4°C-12 h dark cycle for 3 weeks	4	454 sequencer	1,33,077	450	-	DEGs mainly belonged to glycine-rich, cold and drought-regulated proteins, dormancy-associated proteins, proline-rich proteins, and other membrane proteins	Barrios et al., 2017
2	*L. ervoides* (LR-66-637 and LR-66-577)	4 week-old plants leaves/*stemphylium blight* infection/0, 48, 96 and 144 hpi	24	Illumina Hi-Seq 2500	-	1,284	-	Downregulated DEGs were enriched in development-related and energy synthesis-related GO terms and upregulated were enriched in a number of R genes (TIR-NBS-LRR, CC-NBS-LRR genes), cell wall-related and oxidation–reduction processes	Cao et al., 2019
3	ILWL 180, ILL 6002, and 140 RILs	Leaves from 14–21 day-old seedling	142	Illumina Hi-Seq 3000	-	-	4,03,423 SNPs	Seven QTLs with 9.5–11.5% phenotypic variance for Ascochyta blight resistance and presence of 118 SNPs underlying the regions within the intervals of identified QTLs. 44 candidate genes were identified in these QTL regions.	Dadu et al., 2021
4	*L. culinaris* subsp. culinaris (accession “ILL5588” and cv. “Lupa”) and *L. culinaris* subsp. orientalis (BG 16880)	15 day-old seedling aerial part/*Ascochyta lentis* infection/24 hpi	6	Illumina Hi-Seq 2000	50,935	961	-	JA, SA, Auxin, lignin biosynthesis, and chitin response are upregulated in resistant genotypes, while GA pathway was activated in susceptible genotype	García-García et al., 2019
5	Northfield, ILL2024, ILL7537, ILL6788, and Digger, Indianhead	Leaf (young and mature), stem, flowers, immature pods, mature pods, and immature seeds/80 mM NaCl	-	Roche 454 GS-FLX Titanium technology	15,354 contigs and 68,715 singletons	-	2,929 SSRs	166 EST-SSR based markers were amplified in different lentil genotypes of which 51 revealed polymorphism between 12 lentil genotypes	Kaur et al., 2011
6	ILL7537 and ILL6002	14 day-old seedling/*Ascochyta lentis* infection/2, 6, 24 hpi	36	Ion Proton Sequencing	3,17,412	2,617		DEGs included defense related genes at 2 hpi demonstrating early and fast detection of infection, at 6 hpi structural defense responsive genes were activated and at 24 hpi senescence associated gene representing hypersensitive reaction and cell death were observed	Khorramdelazad et al., 2018
7	Cv. Sultan	Root, stem and leaves/drought/1 and 4 day	18	Illumina Hi-Seq4000	2,07,076	2,915 under short-term drought and 18,327 under long-term drought	-	Biological process related to regulation of transcription, DNA-templated transcription, response to abscisic acid, and response to water deprivation were most affected following drought stress	Morgil et al., 2019
8	Genotypes PDL-2 and JL-3	7 day-old seedling leaves/heat stress/35–33°C day-night, 3 h daily for 3 days	12	Illumina Hi-Seq 2000	91,926–1,04,424	~41,000 per comparison	1,41,050 SSRs, 1,94,178 SNPs, and 7,388 Indels	Most of the DEGs were mainly confined to the cell wall and secondary metabolic components	Singh et al., 2019b
9	Cv. Cassab	4 week-old plants-leaf, stem, root, flowers, immature pods, pods, and immature seeds	7	Illumina Hi-Seq 2000	58,986	1,148 tissue specific genes	-	Candidate genes associated with mechanisms of tolerance to both boron toxicity and time of flowering were identified	Sudheesh et al., 2016b
10	Cv. Precoz	20 day-old seedling, root and leaf tissue every 5 days upto 50 days	3	Illumina Genome Analyzer II platform	42,196	2,639–6,719	8,722 SSRs	Pathways specific to root were galactose metabolism, DNA replication and mismatch repair, while the C5-branched dibasic acid metabolism and fatty acid biosynthesis were specific to the leaf	Verma et al., 2013
11	A008, A094, A170, A370, A669, and A677	4 week-old seedling, root, stem and leaves	18	Illumina Hi-Seq platform	2,17,836 transcripts and 1,61,095 unigenes	-	26,449 SSRs and 1,30,073 SNPs	276 EST-SSR markers were verified in 94 lentil accessions of which 125 markers were polymorphic and 43 markers were monomorphic. 127 KASP markers were designed and validation showed that 76 markers were polymorphic, and two were monomorphic	Wang et al., 2020
12	Precoz and WA8649041	Roots, shoots, leaves, branches, and flowers	2	Illumina Hi-Seq 2000	97,528	-	50,960 SNPs	A genetic linkage map having seven linkage groups was generated using 388 SNP, SSR, and ISSR markers	Yilmaz Temel et al., 2015

CC-NBS-LRR, coiled coil nucleotide-binding site leucine-rich repeat; cv., cultivar; d, day; DEGs, differentially expresses genes; EST-SSR, expressed sequence tag simple sequence repeats; GA, gibberellic acid; GO, Gene ontology; h, hour; hpi, hours post-infection; ISSR, inter-simple sequence repeat; JA, jasmonic acid; KASP, Kompetitive allele-specific PCR; MW, molecular weight; RILs, recombinant inbred lines; SA, salicylic acid; SNP, single-nucleotide polymorphism; and TIR-NBS-LRR, Toll interleukin 1 receptor nucleotide-binding site leucine-rich repeat.

Several legumes share similarity in genomes but show different gene expression levels, indicating a diverse difference in terms of biochemical, physical, and developmental nature. Hence, a comparative transcriptome analyses including lentils and other legumes based on exposure of different tissues or cell types to variety of stress can unravel the association between genome and gene function (Garg and Jain, 2013). Plants are often exposed to a combination of stress which greatly impact growth and development rather than a single stress exposure (Suzuki et al., 2014; Abdelrahman et al., 2018). Similarly, lentil faces a combination of stress under field condition and future legume transcriptomic studies, need to be directed toward plants exposed to multi-stress phenomenon. Furthermore, apart from conducting more transcriptomic studies on lentils, an integration of phenomics with transcriptomics, proteomics, and metabolomics is needed to decipher the molecular mechanisms representing lentil development under stresses.

## Proteomics an Emerging Area for Lentil Improvement

Genomics and transcriptomics have emerged as major techniques that facilitated the identification of candidate loci or gene/s, which can be utilized for crop improvement. A parallel evolution of analytical instrumentation and bioinformatics techniques has allowed proteomics to emerge as a pioneer tool. Proteomics reveals downstream translational and post-translational dogma of biochemical pathways and molecular nexus underlying plant biorhythm, environmental interactions, and responses to varying stresses (Hu et al., 2015). High-throughput proteomics expanded its realm from protein, protein–protein interactions, and protein complexes identification to quantitative profiling, dissection of post-translational modifications (PTMs), signaling pathways, and prediction/validation of subcellular localization (Hu et al., 2015). The protein translation and PTMs are major response mechanisms activated for immediate molecular responses to provide stress adaptation (Ghatak et al., 2017). Hence, it is imperative to quantify protein levels and identify protein undergoing post-translational modifications to decipher stress-inducible signaling processes. Thus, proteomics has emerged as one of the facets for dynamic analysis of molecular changes at the protein level which are not captured by genomics or transcriptomics approaches.

A two-dimensional electrophoresis analysis of mature lentil seeds was performed by Scippa et al. (2008) in 17 different lentil populations, revealing 193 resolved protein spots, of which 71 were differential proteins within the populations. A similar investigation by Scippa et al. (2010) on seed proteome of 12 lentil populations identified 122 proteins by employing MALDI-TOF PMF and/or nanoLC-ESI-LIT-MS/MS technique, including 103 differentially expressed proteins. Further, among these, 24-protein species could be used to differentiate populations and hence considered as potential markers. Seven different Italian lentil seeds (*Giganti*, *Mignon*, *Piccole*, *Rosse Intere*, *Verdi*, *Rosse Decorticate*, and *Nere*) were examined by Caprioli et al. (2010) using a MALDI-TOF Ultraflex II to obtain protein fingerprints. The relative *m/z* values or spectral complexity (such as ionic species present at *m/z* 5,530, 5,621, 5,877, 7,574, 12,070, 13,285, and 13,450) obtained by the MALDI spectrometer was utilized to perform hierarchical clustering analysis. Clustering revealed *Verdi* behaving differently in terms of *m/z* values compared to other varieties followed by *Rosse Intere*, *Giganti*, and *Nere*, whereas *Rosse Decorticate*, *Piccole*, and *Mignon* were quite similar (Caprioli et al., 2010). This investigation showed that spectral values are an effective method to differentiate lentil varieties. A recent study on low-molecular weight protein profiling in seven different lentil cultivars by Shaheen et al. (2019) identified 2,873 peptides associated with 180 unique proteins. This study revealed cultivar-specific allergen proteins in lentils.

We performed an independent analysis in this review to identify a common set of proteins from these seed proteomes. For this, a non-redundant set of proteins were obtained from these two studies (387 proteins, Shaheen et al., 2019; and 63 proteins, Scippa et al., 2010) and then compared for the presence of common protein families/proteins (Figure 2D). We identified 43 common proteins belonging majorly to the seed storage protein family, such as vicilin, convicilin, allergen, lectin, legumin, P54 protein, and albumin (Supplementary Table 2). This analysis demonstrates that proteomic studies can be used to screen allergens in different cultivars. Moreover, cultivars with the lowest level of allergens can be selected for molecular breeding programs to develop nutritionally enriched, allergen deprived high-yielding lentil varieties.

Plant traits involve an intricate interplay between posttranscriptional, translational, or post-translational regulation. Protein QTLs (pQTL) identification can envisage the result of genetic crosses (Li et al., 2018). Proteomics can be used to identify pQTLs in lentils as performed in mouse strains (Chick et al., 2016). Similar proof of concept has been applied in potato (*Solanum tuberosum*), where Chawade et al. (2016) showed that selected reaction monitoring (SRM, a targeted mass spectrometry) based proteomics can assist in precision breeding. Briefly, Chawade et al. (2016) predicted 104 potato peptides based on univariate and multivariate statistics followed by application of random forest classification approach to identify markers for *Phytopthora infestans* resistance. Targeted proteomics can also be used to study phosphoproteomics in lentil. Van Ness et al. (2016) combined iTRAQ (protein relative quantification) and SRM assays (targeted proteomics) to identify five phosphopeptides which show rapid phosphorylation increase within 1 h of Nod factor treatment in *Medicago truncatula*.

Proteomic technologies have undergone an upgradation from gel-based assays to gel-free assays and label-free proteomics (Jan et al., 2022). Lentil proteomics need to shift from conventional to modern proteomics approach to not only identify candidate proteins but also corresponding protein PTM sites in a time-efficient and high-throughput manner. Application of these technological innovations in lentil breeding is needed to breed tolerant/resistant varieties. In the case of lentils, only limited proteomics studies have been performed to date, leaving a wide gap in proteomic information for breeding use and inaccurate predictions. In addition, no studies have been conducted to quantify protein levels or post-translational modifications under biotic or abiotic stresses. Therefore, conducting proteomics studies to identify donors with differential protein levels is critical and timely. The identified donors can be used for lentil breeding for developing lentils with enhanced tolerance that are better adapted to future climatic conditions.

## Metabolomics: An Underutilized Technique in Lentil Breeding

Recent breakthroughs in sequencing platforms have resulted in unprecedented information that provides access to minuscule levels of genomic information in crop plants (Matasci et al., 2014).^2^ Metabolome is the apogee of omics technologies representing the endpoint of information flow and corresponds strongly to the phenotype. Plant metabolites are further categorized as primary and secondary metabolites and are the functional outcome of cellular regulatory mechanisms. Metabolic pool comprises either reactants, products, or even the intermediates of enzyme-catalyzed or chemical reactions running in a biological system (Liu and Locasale, 2017). Their cellular concentrations indicate the response of biological machinery to corresponding genetic or environmental interactions (Fiehn, 2002; Mishra et al., 2017, 2020).

An interspecies comparative study for bioactive compounds was conducted by Llorach et al. (2019) between chickpea, lentil, and white bean using Liquid Chromatography-Mass Spectrometry-Orbitrap metabolomic technique. The study revealed 43 differential compounds (belonging to six classes of phytochemicals: prenol lipids, polyphenols, fatty acyls, organic compounds, α-galactosides, and nucleosides) between the three legumes of which 40% were exclusive to lentils (Llorach et al., 2019). Lentils were distinguished from the other two legumes majorly in terms of flavanol compounds, as virtually all identified flavanol compounds belonged exclusively to lentils with remarkable differences compared to other legumes. The analysis also showed that megastigmadiene-diol-[apiosyl-glucoside] and resveratrol glucoside were also discriminately present in lentils. Additionally, a similar interspecies comparative study involving comparison of lentil and *Lupinus* using liquid chromatography-mass spectrometry-based metabolomics study performed by Farag et al. (2019) identified 66 metabolites. Some of the metabolites identified were sphingolipids, flavonoids, saponins, fatty acids, phenolics, and alkaloids. Metabolome profiling revealed saponin and flavone glycosides as species-specific biomarkers for lentils, whereas *Lupinus* seeds have a higher abundance of flavanone glycosides. These comparative interspecific studies help distinguish legumes for nutritional consumption based on the type of metabolic compounds produced and the quality control of food.

Similar to transcriptome and proteome, we performed independent analysis in this review to identify a common set of metabolites in these two studies. We sorted the non-redundant metabolites based on their molecular formula (43 from Llorach et al., 2019 and 94 from Farag et al., 2019; Figure 2E). We found 19 metabolites to be commonly present, which belonged to classes: flavonol/flavone/flavan derivatives, terpene glycoside, phenolic acid, organic acid, and saccharides (Supplementary Table 3).

Apart from interspecies metabolite screening, intraspecies global metabolite profiling has been performed in lentils. One such investigation performed by Skliros et al. (2018) involved two salt-stressed lentil varieties from different geographies. The salt stress resulted in low levels of organic acids, accumulation of sugars and polyols in leaves, and other key metabolites, such as urea, L-asparagine, allantoin, and D-trehalose, in the roots (Skliros et al., 2018). The presence of polar metabolites was a qualitative and quantitative distinction factor between root and leaf as well as within the two varieties. These comparisons indicate organ and variety-dependent metabolite responses. A similar investigation to examine phenotypic and metabolic attributes linked to drought and/or salinity stress tolerance was carried out by Muscolo et al. (2015) involving metabolite profiling using GC–MS in four accessions of lentils-Castelluccio di Norcia (CAST), Eston (EST), Pantelleria (PAN), and Ustica (UST). Upon polyethylene glycol (PEG) treatment, the polyamine content increased in PAN and UST compared to EST and CAST, and a significant decrease in organic acids was also noticed. Further, majority of amino acids showed increased levels in genotype treated with PEG. A low level of proline was observed in NaCl-treated UST and PAN compared to EST and CAST. The metabolites showing significant variation compared to others under stress were trehalose, maltose, proline, and isoleucine (Muscolo et al., 2015). The outcome of these examinations in lentils can be summarized as a transient metabolic adjustment in response to stress, and the extent of response depends upon the type, growth stage, and severity of stress. Additionally, besides known abiotic stress marker metabolites, drought-specific marker metabolites ornithine and asparagine and salinity-specific markers alanine and homoserine were also identified. Interestingly, these studies showed strong organic acid depletion after exposure to salinity stress (Muscolo et al., 2015; Skliros et al., 2018). This result reflects low carbon availability and hence a metabolic shift against the formation of high-carbon molecules (Sanchez et al., 2008).

Again, we performed an analysis to identify non-redundant metabolites in two studies of drought/salinity (72) and salinity (258; Figure 2F). Based on our analysis, 31 common metabolites were identified in these drought and salinity studies, belonging to polyamines, organic acids, sugars and polyols, and amino acids (Supplementary Table 3). These common set of metabolites can be potential markers that can be targeted for breeding combined abiotic stress tolerance in lentils.

A new paradigm in the form of metabolomics has emerged as a potential approach to establish signature metabolites as markers associated with agronomically important traits (Schaarschmidt et al., 2021). Metabolite levels are a quantitative trait and hence in QTL mapping and GWAS they are considered as quantitative phenotypic trait. Genomic mapping of metabolites using metabolite-based QTL mapping (mQTL) and GWAS (mGWAS) is widely used, but not a single study has been conducted in lentils till date (Litvinov et al., 2021). Therefore, metabolomics-assisted breeding needs to be widely explored for efficient screening and selection of breeding material for improving yield and enhanced stress tolerance in lentils (Kumar et al., 2018c). An integration of metabolomics and recent genomics tools, such as whole-genome sequencing, GBS, and genome-wide genetic variants, provided strong evidence for dissection of genetic and phenotypic association in crop plants.

## Modern Breeding Approach Using Multi-Omics Data Integration: Future of Lentil Breeding

**Figure 3:**
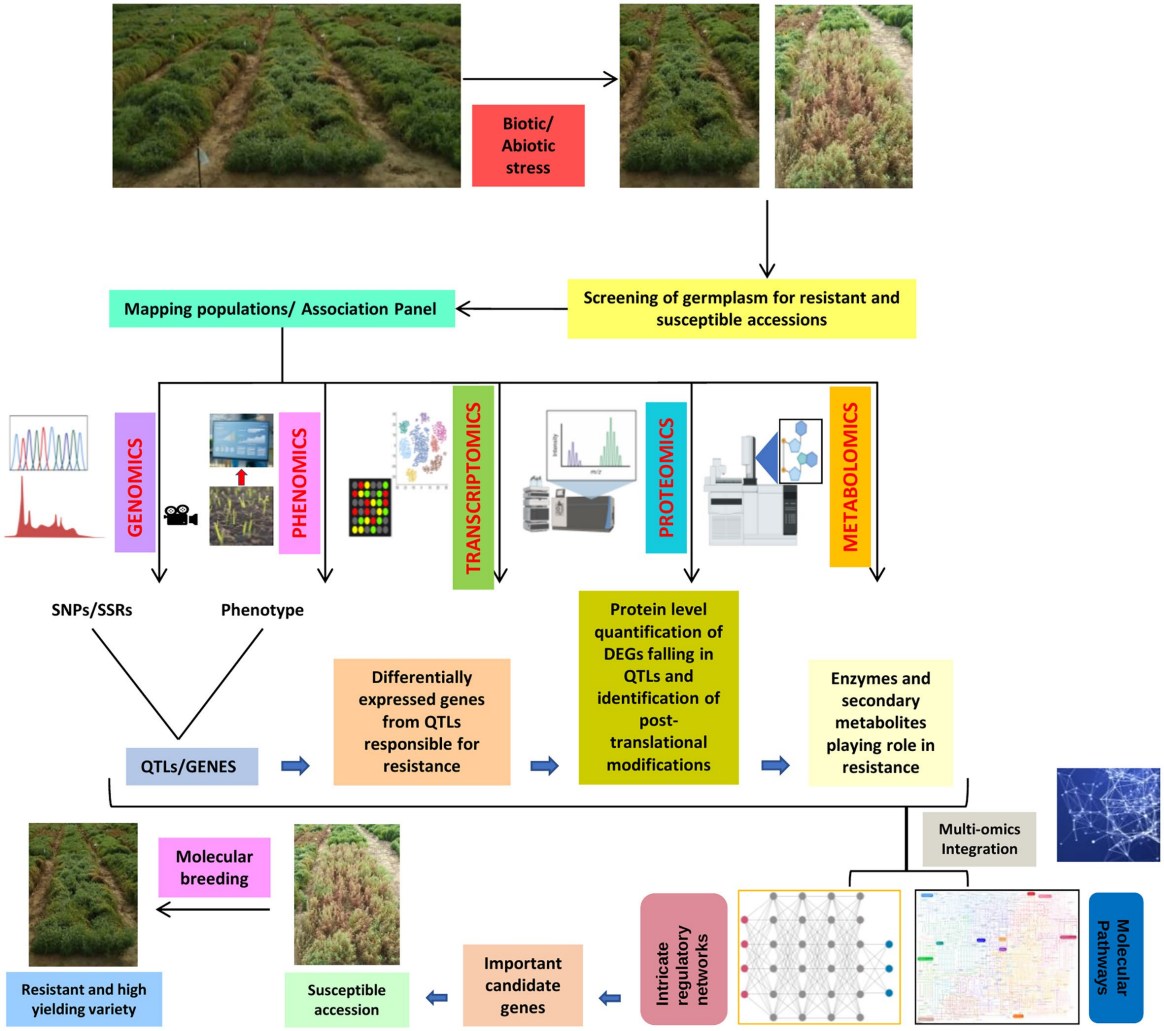
A diagrammatic scheme for integration of omics approach (multi-omics integration, MOI) for lentil crop improvement.

The integration of genomics, transcriptomics, proteomics, metabolomics, and phenomics involves a modern field of system biology that offers a holistic rather than reductionist approach to explore the biology behind complex morpho-physiological semblance upon interaction with biotic and abiotic stresses (Figure 3; Aizat et al., 2018). The major limitation faced by omics research includes proper handling of biological/machine/human interface for data processing and integration (Palsson and Zengler, 2010). For the application of the multi-omics integration (MOI) approach in lentils, we need a planned scientific methodological approaches, such as the ones implemented in mixOmics, Plant Regulomics, PaintOmics 3, etc., to extract the information from different omics technologies, combine the results obtained, backed by critical thinking to merge different data sets (Rohart et al., 2017; Hernández-de-Diego et al., 2018; Wang et al., 2018; Ran et al., 2020). Therefore, future studies need to largely aim at MOI to help address the gap between data generation and correlation for better understanding the intrinsic mechanisms lentils adopt in response to environmental cues. The MOI can be implemented by combining results obtained from different omics techniques and modeling approaches to determine complex traits and phenotypes. Efforts are already being taken to integrate different omics data to carve out logical explanations such as genomics- and phenomics-based GWAS to establish a relationship between genomic features such as QTLs or SNPs or MTAs (Marker-Trait Associations) with resulting phenotypes. Similarly, the study can be broadened to identify candidate genes in the QTL region, their transcription and expression profile by performing a transcriptome analysis, the translation efficiency in capturing protein abundance by performing proteomics investigations, and also recognizing the role of any secondary metabolites if present by metabolomics (Figure 3). The same methodology can be used at a bigger scale to decipher complete pathways starting from the gene to transcript, protein, metabolite, and resulting phenotype. A well-formulated MOI strategy for developing high-yielding, climate, and disease resilient lentil cultivars can be extended to other less-studied crops plagued with extremely low productivity.

The data available through omics approaches can also be used to bridge the gap in conventional breeding. Several strategies which need immediate attention to improve lentil productivity includes, mutation breeding, clustered regularly interspaced short palindromic repeats (CRISPR), and CRISPR-associated (Cas) based genetic dissection, speed breeding, and shuttle breeding. Mutation occurs naturally at a very slow pace to produce variants which can carry enhanced traits. Spontaneous mutation taking place in nature can take several years to produce desired phenotype. Mutations can be induced to create novel variants either by chemicals (ethyl methanesulfonate), radiations (gamma rays, X-rays, or ion beams), and insertional mutagenesis (T-DNA or transposons; Oladosu et al., 2016). Successful mutation breeding primarily involves identification of advantageous variant mutants in the second or third generations. Thus, effective mutant breeding relies heavily on accurate genomic information and sound high-throughput phenotyping technologies. Target-induced local lesions in the genome (TILLING) and Eco-TILLING can be used to screen mutation in the target genes (Wang et al., 2012; Singh et al., 2019a). Sequencing-based approaches such as genome resequencing, bulked segregant analysis, association mapping, and fine gene mapping revolutionized germplasm improvement and genome manipulation through identification of single base-pair polymorphisms such as SNPs, SSRs, and QTLs (Edwards and Batley, 2010; Bolger et al., 2014; Zou et al., 2016). Marker-assisted selection is preferred over traditional breeding as it involves tracking of mutations responsible for enhanced backcrossing efficiency and determination of progeny phenotypes homogeneity (Nadeem et al., 2018).

Accurate genomic information can be utilized for plant genome editing. Sequence-specific nucleases (*SSNs*) such as CRISPR-Cas9 system and its variants can perform plant genome editing to produce stably inherited and desired gene modification (Nekrasov et al., 2017). CRISPR-Cas9 system is widely used technique to produce transgene-free desired phenotype. The popularity lies in it being easy to operate, cheap, and time-efficient, with a high success rate (Satheesh et al., 2019). It involves a short-guided RNA (sgRNA) directed toward gene to be edited and an RNA-guided DNA endonuclease Cas9 complex (Belhaj et al., 2015). Rodríguez-Leal et al. (2017) reported use of Cas9-induced mutagenesis for fine-tuning of quantitative traits by targeting promoter regions. Utilizing a multitude of sgRNAs simultaneously directed toward several cis-regulatory elements, they generated a heterogeneous population with different alleles in tomato and slightly different phenotypes. Therefore, not only knock-out of genes but also Cas9-mediated genome engineering has moved forward to genomic manipulations like fine-tuning of quantitative traits. Similarly, genomics, transcriptomics, proteomics, and metabolomics approaches in lentils can be used to identify promoter/gene/protein/metabolites of interest responsible for particular trait and the required gene can be edited *via* CRISPR to produce resistance or tolerant varieties. CRISPR-Cas9 system application in lentil has not been achieved till date and researchers could focus on implementation of genome editing *via* CRISPR to produce improved lentils.

All breeding approaches or genome editing tools suffer due to long breeding cycle and an acceleration in crop research and improvement demanded rapid generation advancements. Speed breeding has emerged as a novel technique which can shorten the harvest time and can generate up to six generations in a year (Ghosh et al., 2018). The technique depends on extension of photoperiod length and therefore has been successfully implemented in short-day plants such as spring wheat (*Triticum aestivum*), grass pea (*Lathyrus sativus*), barley (*Hordeum vulgare*), *Brachypodium distachyon*, canola (*Brassica napus*), and chickpea (*Cicer arietinum*; Ghosh et al., 2018). Successful application of speed breeding in lentils can provide a boost to lentil research programs by saving time in studying phenotypes of traits, mutants, and transformation generated from other breeding techniques. This can drastically reduce the duration to develop new crop varieties. Speed breeding can be associated with shuttle breeding to produce improved varieties at a rapid pace. Shuttle breeding concept was introduced by CIMMYT (International Maize and Wheat Improvement Center). This breeding approach uses diverse climatic conditions to generate enhanced and highly adapted varieties (Ortiz et al., 2007). Briefly, the approach requires optimum testing and selection environments to record yield. Segregating populations are grown in variable environments and simultaneously selected and advanced lines are screened at distinct sites. This results in identification of superior and improved genotypes, which shows resistance to diseases and tolerance to different types of stresses (Ortiz et al., 2007). A similar approach can be used in lentils by growing them in diverse conditions to develop tolerant varieties. Different omics approaches can be applied to the superior genotype to identify the molecular mechanism underlying the superiority of genotype under diverse climate and the same can be extended to other crops.

In conclusion, it can be inferred that the lentil crop is lagging in the application of modern omics technologies at every aspect particularly, proteomics and metabolomics compared to other crops. There is a huge potential for lentil improvement through application of omics studies and their data integration to produce climate and biotic stress resilient high-yielding lentil cultivars.

## Author Contributions

MT and SJ conceived and prepared the outline of review. MT and BS analyzed the data and performed the analysis. MT, BS, DM, and SJ wrote the paper. MT prepared all the figures and tables. All authors contributed to the article and approved the submitted version.

## Conflict of Interest

The authors declare that the research was conducted in the absence of any commercial or financial relationships that could be construed as a potential conflict of interest.

## Publisher’s Note

All claims expressed in this article are solely those of the authors and do not necessarily represent those of their affiliated organizations, or those of the publisher, the editors and the reviewers. Any product that may be evaluated in this article, or claim that may be made by its manufacturer, is not guaranteed or endorsed by the publisher.

## Footnotes

^1^ http://knowpulse.usask.ca

^2^ https://db.cngb.org/10kp/
